# Application of radiomics model based on FDG-PET/CT for the assessment of therapeutic effect in patients with newly-diagnosed multiple myeloma

**DOI:** 10.3389/fonc.2025.1647730

**Published:** 2025-12-01

**Authors:** Fukai Li, Fei Li, Xiaodan Xu, Xiang Wang, Qingyang Yu, Guangwen Duan, Jiayang Yan, Baiyang Jiang, Hongbiao Sun, Shaochun Xu, Kaili Chen, Yi Xiao, Shiyuan Liu

**Affiliations:** 1 Department of Radiology, Second Affiliated Hospital of Naval Medical University, Shanghai, China; 2 Department of Research and Development, Shanghai United Imaging Intelligence Co., Ltd., Shanghai, China; 3 Department of Hematology, School of Medicine, Shanghai Fourth People's Hospital, Tongji University, Shanghai, China

**Keywords:** multiple myeloma, therapeutic assessment, radiomics, PET/CT, deep response

## Abstract

**Objectives:**

To evaluate the prediction value of radiomics models based on FDG-PET/CT for the therapeutic effect in patients with newly-diagnosed multiple myeloma (MM).

**Materials and methods:**

We retrospectively reviewed the clinical characteristics and ^18^F-FDG-PET/CT imaging data of 165 MM patients. Randomly divided into a training set (n=133) and a test set (n=32) at a ratio of 8:2. All patients underwent whole-body PET-CT scans within one month prior to the commencement of treatment. Overall response rate was the principal efficacy endpoint, including stringent complete response (sCR), complete response (CR), very good partial response (VGPR), partial response (PR), disease stabilization (SD), and disease progression (PD). Deep response (DR) was defined as sCR, CR, and VGPR, while non-deep response included PR, SD and PD, 74 patients attained DR. Different models involving clinical, radiomics extracted from PET/CT, and their combination were constructed based on multiple logistic regression and logistic regression machine learning classifier after features selection, respectively. The models predicting performance were evaluated by the area under the ROC curve (AUC), sensitivity, specificity, accuracy, precision, and F1 score. Receiver Operating Characteristic (ROC) curves, decision curves, calibration curves, and DeLong’s test were applied to compare their ability.

**Results:**

Gender was the only one of clinical characteristics found to be independent prognosis factor for treatment evaluation, with a p-value of 0.041. The radiomics models outperformed the Clinical model significantly, among which the PET-CT model yielded the best results with the AUC of 0.809. The PET + CT + Clinical model achieved the optimal performance after integrating clinical and radiomic features, with the AUC of 0.813.

**Conclusions:**

The FDG-PET/CT-based radiomics model, particularly when integrated with clinical features, can more effectively predict deep treatment response in newly diagnosed MM patients, offering significant clinical utility for early treatment stratification and personalized therapeutic guidance.

## Highlights

- Radiomics with 18F-FDG PET/CT imaging to assess treatment efficacy in newly diagnosed MM patients.
- The clinical and radiomic data to distinguish between multiple myeloma patients demonstrating deep response to treatment and those exhibiting non-deep response.
- Radiomics model based on PET/CT features significantly outperformed models based solely on clinical or PET characteristics.

## 1 Introduction

Multiple myeloma (MM) is an incurable malignancy characterized by clonal plasma cell proliferation in the bone marrow and excessive monoclonal immunoglobulin production (1). MM constitutes 1.8% of all cancer cases, with an annual incidence rate of 4.6 to 6 cases per 10,000 individuals, thereby ranking as the second most prevalent hematologic malignancy (2, 3). In the past two decades, the overall survival rate for MM patients has significantly improved due to the introduction of novel therapeutic agents (4, 5). Concurrently, the development of sensitive methods for measuring deep therapeutic responses has led to the concept that sustained minimal residual disease (MRD) negativity is associated with remarkably low progression rates (6–8). However, despite these unprecedented treatment responses, 30-50% of patients fail to achieve or maintain MRD negativity, ultimately progressing to advanced stages of the disease (4). Moreover, in clinical practice, significant disparities in survival outcomes are observed among patients presenting with the same disease stages (9). Current prognostic staging systems have limitations in accurately stratifying risk, particularly for intermediate-stage disease (10, 11). Understanding the therapeutic effects and prognostic characteristics of patients with MM is essential for optimizing treatment strategies.

Despite advances in the treatment of MM, relapse remains common. Furthermore, there are no standard treatments for patients with advanced MM following primary therapies; complete responses are rare, with a median progression-free survival of 3–4 months and an overall survival of 8–9 months (10). Current assessment methods are often invasive, time-consuming, and difficult for some patients to undergo. Due to the wide variability in survival among MM patients, developing a more accurate and comprehensive therapeutic assessment system remains a major focus and challenge in clinical practice.

Accurate therapeutic effects and prognostic stratification are essential for selecting appropriate treatment strategies for MM, so as to prevent overtreatment and reduce the medical and financial burden. The International Myeloma Working Group recommends 18F-FDG PET/CT as one of the preferred imaging modalities for evaluating MM and other plasma cell disorders (3, 11). PET/CT integrates both anatomical and metabolic information, providing high sensitivity and specificity for assessing bone damage and detecting extramedullary disease (3). It can identify additional lesions in up to 40% of patients with early-stage MM (12). Compared with MRI and X-ray, PET/CT has superior capabilities for detecting extramedullary disease, making it an indispensable tool in the diagnostic and therapeutic assessment of MM (13). Many studies have indicated that imaging features such as focal lesions, metabolic activity, tumor volume, and extramedullary disease are associated with survival outcomes (14) However, most studies focus solely on imaging features, overlooking the integration of clinical, laboratory, and cytogenetic data for a comprehensive assessment. Moreover, current evaluations still largely depend on physicians’ visual interpretation and basic quantitative metrics such as lesion count, SUVmax, metabolic tumor volume, etc., which are subject to interobserver variability and inconsistent definitions. This underscores the need for more robust, reproducible, and integrative assessment methods.

Radiomics has emerged as an important field involving the automated or semi-automated extraction of quantitative features, including texture, intensity, and density, from medical imaging (15). Numerous studies have demonstrated the efficacy of radiomics in the differential diagnosis of cancer, the prediction of treatment response, and the prognosis of disease progression (16–18). In our prior studies, we successfully employed radiomics models based on MRI to differentiate between spinal MM and metastases (19, 20). Although radiomics studies based on PET/CT are gaining attention, their application in assessing treatment response for MM remains limited, mainly focusing on predicting overall survival or recurrence (21–26).

This study aimed to investigate the feasibility of utilizing radiomics based on 18F-FDG PET/CT imaging to evaluate the therapeutic efficacy in patients newly diagnosed with MM. Additionally, the research sought to develop a model integrating clinical and radiomic data to differentiate between MM patients exhibiting deep response and those with non-deep response to treatment.

## 2 Materials and methods

### 2.1 Study population and inclusion and exclusion criteria

This retrospective study was approved by the Ethics Committee of Changzheng Hospital of the Navy Medical University (No.2016SL019A), and the informed consent was waived. From December 2015 to December 2022, we collected clinical and PET/CT information of MM patients. Each patient was diagnosed with MM through comprehensive histological and hematological examinations, in accordance with the IMWG guidelines for both diagnosis and treatment (1). The inclusion criteria were: (1) diagnosed according to the IMWG diagnostic criteria; (2) complete pre-treatment and post-treatment clinical data; (3) hospitalized and received two courses of standard induction chemotherapy. The exclusion criteria were: (1) combined malignant tumors or hematological diseases in other systems; (2) concomitant cardia amyloidosis; (3) chemotherapy and radiation therapy before the PET/CT examination; (4) poor image quality. Full-body PET/CT examinations were conducted within one month before the initiation of treatment for each patient.

Based on the inclusion and exclusion criteria, a total of 165 patients (90 males and 75 females) were enrolled, with a median age of 61 years and an age range between 34 and 86 years. All patients underwent comprehensive blood-based laboratory tests. Laboratory indicators encompassed M protein, sFLC (free light chain), hemoglobin, creatinine, albumin, Ca2+, lactate dehydrogenase, β2-microglobulin levels, platelets, hypersensitive C-reactive protein, and PET/CT quantitative parameters. Serum protein, serum albumin, glucose filtration rate, beta-2 microglobulin, hemoglobin, hematocrit, calcium levels, and serum lactate dehydrogenase were additionally quantified.

### 2.2 Treatment and response evaluation

The treatment plans are divided into three categories: chemotherapy regimens based on proteasome inhibitor–based therapy (PI-based), chemotherapy regimens based on immunomodulatory drug-based therapy (IMiD-based), and chemotherapy regimens based on the combination of proteasome inhibitor-containing therapy and immunomodulatory drug-based therapy (IMiD+PI).

The principal efficacy endpoint was the deep response rate (DR), defined as the proportion of patients achieving at least a very good partial response (VGPR), complete response (CR), or stringent complete response (sCR), according to Paiva B et al. and the International Myeloma Working Group (IMWG) criteria (27, 28). For descriptive purposes, we also calculated the overall response rate (ORR), defined as the proportion of patients achieving partial response (PR) or better (PR, VGPR, CR, sCR). Patients achieving sCR, CR, or VGPR were categorized as DR, whereas those with PR, minimal response (MR), stable disease (SD), or progressive disease (PD) were categorized as non-DR for subsequent binary classification analyses.

**Figure 1:**
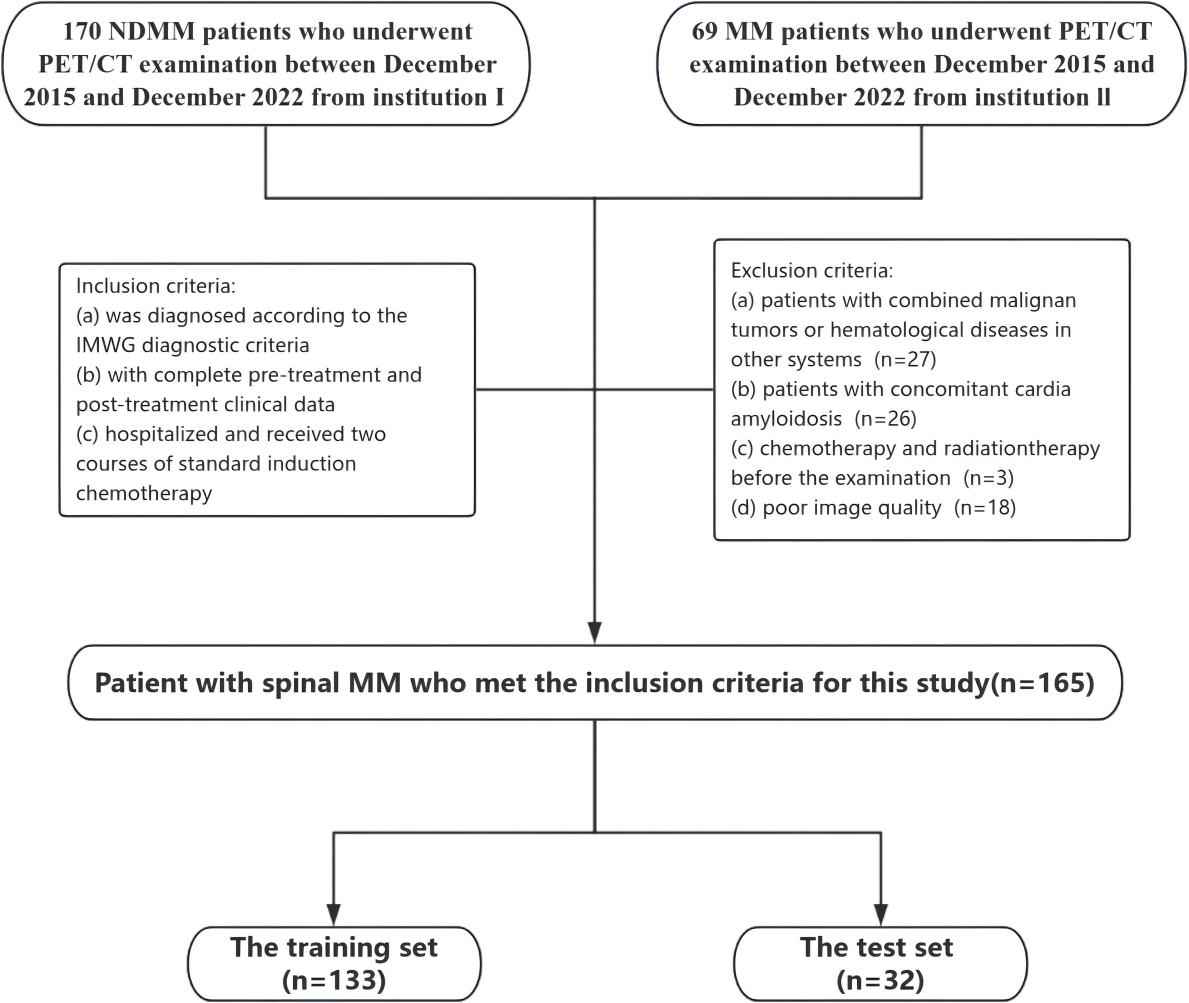
Flowchart summarizing patient enrolment process and study cohorts.

MRD testing was performed within two weeks of the first confirmed CR using multiparameter flow cytometry (MFC) with a sensitivity of 10^^-4^, following IMWG recommendations. MRD status was recorded as positive or negative. All treatment response assessments were conducted by expert hematologists. Baseline characteristics and treatment details are provided in **Table 1**, and the study design is outlined in **Figure 1**.

**Table 1:** Basic patient information of training set and testing set.

Variables	Training set (n=133)	Testing set (n=32)	P-values
Age (mean±sd)	58.722 ±8.847	61.062 ±8.810	0.181
Gender (0/1)			
Male	72	18	0.829
Female	61	14	
Initial treatment plan			
PI-based	97	25
IMiD-based	8	2	0.725
PI+IMiD	28	5	
Bone marrow plasma cell (≥60%)			
Yes	28	2	0.051
No	105	30	
FL(≥3)			
Yes	109	28	0.453
No	24	4	
EMD			
Yes	60	14	0.889
No	73	18	
HB (≤100g/L)			
Yes	60	15	0.96
No	73	17	
Cr (≥177umol/L)			
Yes	24	6	0.926
No	109	26	
ALB (≥35g/L)			
Yes	65	12	0.247
No	68	20	
LDH(≥250U/L)			
Yes	16	5	0.584
No	117	27	
β2-MG (≥5.5mg/L)			
Yes	33	7	0.728
No	100	25	
PLT (<100*10^9/L)			
Yes	13	2	0.534
No	120	30	
HCRP (>10mg/L)			
Yes	30	4	0.207
No	103	28	
Ca2+ (≥2.55mmol/L)			
Yes	31	5	0.345
No	102	27	
DS.staging (1/2/3)			
I	12	3	0.77
II	26	8	
III	95	21	
ISS.staging			
I	34	4	0.167
II	42	15	
III	57	13	
R-ISS.staging (1/2/3)			
I	27	5	0.494
II	18	18	
III	25	9	
Liver SUVmax (Median[Q1~Q3])	2.09[1.82,2.40]	2.085 [1.740,2.620]	0.995
SUVmax(Median[Q1~Q3])	5.500 [4.200,7.480]	5.335 [3.72,7.33]	0.688
TLG(Median[Q1~Q3])	39.0 [20.0,78.0]	44.5 [13.8,143.3]	0.493
MTV(Median[Q1~Q3])	12.0 [6.0,24.0]	13.0 [5.75,32.25]	0.462

PI-based, Proteasome inhibitor-based; IMiD-basedimmunomodulatory drug-based; FL, focal lesion; EMD, extramedullary; HB, hemoglobin; Cr, creatinine; ALB, albumin; LDH, lactate dehydrogenase; β2-MG, β2-microglobulin; PLT, platelet; HCRP, hypersensitive C-reactive protein; DS staging, Durie Salmon staging; ISS, International Staging System; R-ISS, the revised International Staging System; SUVmax, max standardized uptake value; TLG, total lesion glycolysis; MTV, metabolic tumor volume.

### 2.3 Equipment and parameters

All images were obtained from the hospital’s Picture Archiving and Communication System (PACS) and scanned using the SIEMENS Biograph 64-layer PET/CT equipment. Patients underwent a fasting period of over 6 hours before being injected with 18F-FDG at a concentration of 0.15-0.18 mCi/kg. Typically, the PET/CT scan commenced 60 minutes post-injection. Patients were positioned supine with both upper arms placed above their heads to minimize chest artifacts.

The procedure began with a body CT scan, using scanning parameters of tube voltage of 120 kV, tube current of 150 mA, layer thickness of 3mm, and scanning range from the top of the skull to the middle of the femur. The body PET/CT scan was collected for 5–6 beds, with a conventional collection time of two minutes per bed. Subsequently, CT data was utilized for attenuation correction and PET image enhancement, followed by image reconstruction and fusion.

### 2.4 PET image delineation and registration between PET and CT images

Two experienced radiologists, with 6 and 7 years of expertise respectively, conducted blind segmentation of lesions exhibiting the highest uptake on PET scans. In cases of discrepancies concerning PET/CT findings, a consensus was reached through a collaborative review involving a senior nuclear medicine physician with over 10 years of experience. The interpretation of PET images adhered to IMWG standards, defining focal lesions as those exhibiting higher uptake than the hematopoietic bone marrow background (BM) or liver, with a minimum diameter of 5 mm. Diffuse uptake was defined as uptake above that of the liver (29).

Evaluation of images was carried out by a team of experienced nuclear medicine physicians. following established criteria for assessing myeloma lesions. Briefly, positive areas were indicated by the presence of focal areas with increased tracer uptake within bones (SUV ≥2.5), with or without any underlying lesions identified on CT or osteolytic CT areas >0.5 cm (11). The Metabolic Tumor Volume (MTV) MTV was calculated from PET data of the delineated volume. To enhance segmentation consistency, two radiologists randomly selected 20 patients for intra- and inter-observer consistency tests. Intra-group and inter-group consistency coefficients (ICC) between features were computed to identify and retain features demonstrating robust repeatability (ICC>0.70).

**Figure 2:**
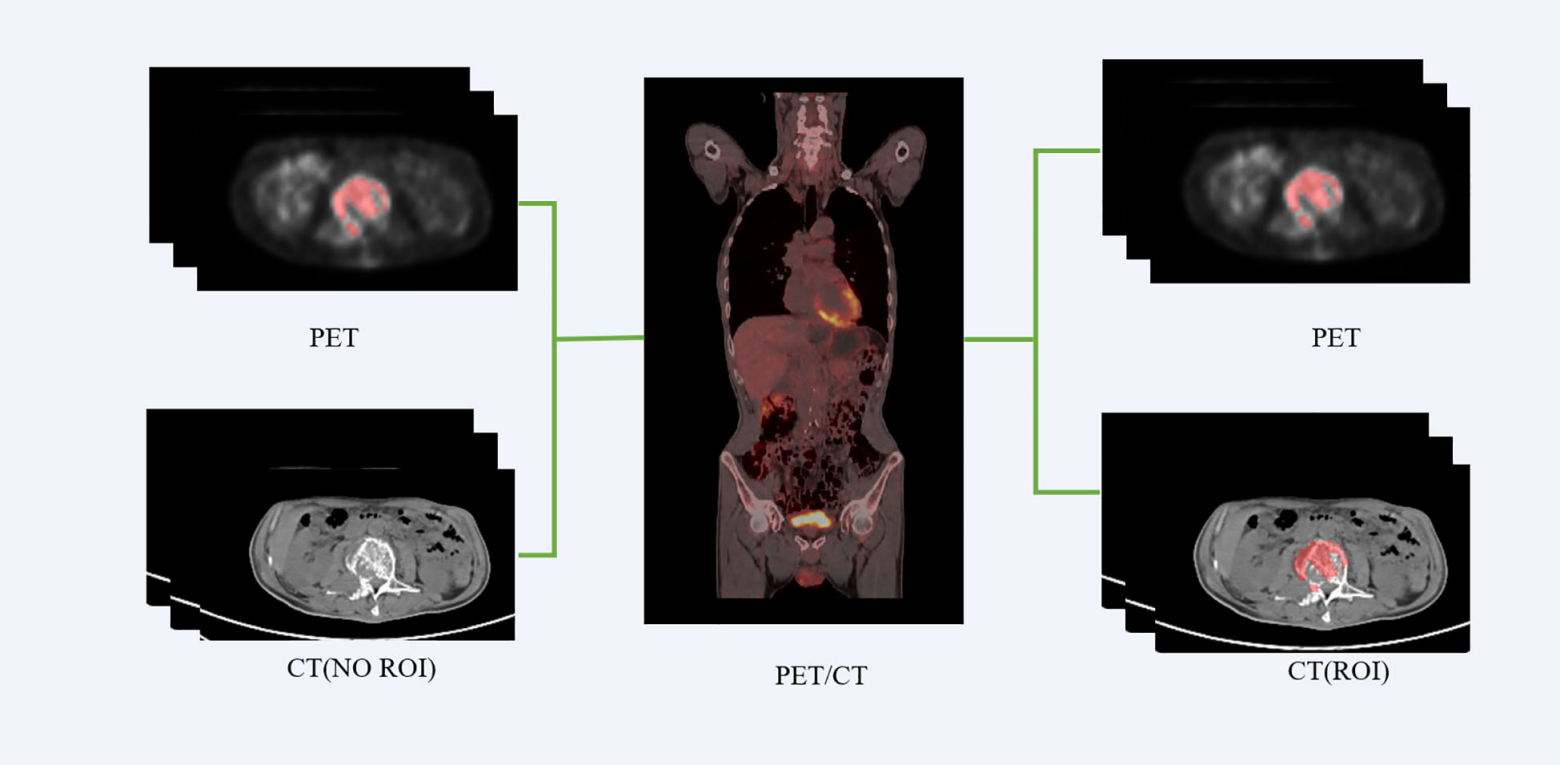
PET-CT registration process flowchart.

We employed the PET/CT registration method available on the platform (https://www.uii-ai.com/research.html) for the automated alignment of PET and CT images. Subsequently, a senior medical radiologist reviewed the registered images to confirm the precise alignment of major organ boundaries, such as the skin, skeletal structures, and liver. A registration matrix was generated to quantify this alignment. Ultimately, the regions of interest (ROI) identified from the PET images were overlaid onto corresponding locations within CT images (**Figure 2**).

### 2.5 Radiomics extraction and selection

**Figure 3:**
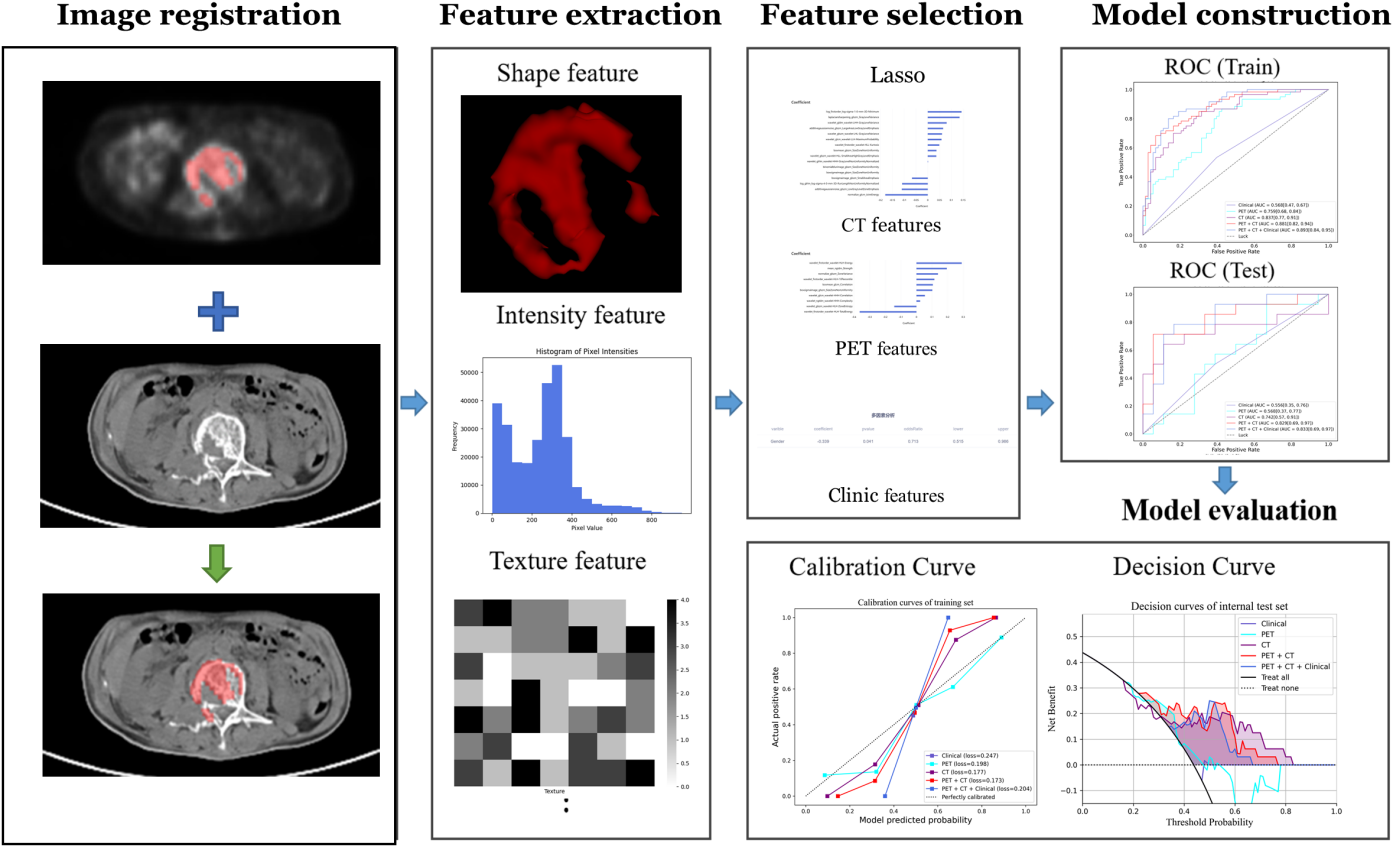
Radiomics workchart for response evaluation in MM.

Radiomics analysis was performed by a clinical research platform (uAI Research Portal, United Imaging Intelligence Co., Ltd, China). Radiomics features were extracted from these images using the PyRadiomics toolbox in Python 3.7. The flowchart of the radiomics analysis is shown in **Figure 3**. Initially, all parametric maps underwent normalization using maximum and minimum truncation processing. Subsequently, 14 image filters were used to generate derived images, from which first-order statistics and texture features were extracted, resulting in a total of 2,160 derived features. From the largest focal area of myeloma in each patient, 2,264 radiomics features were automatically extracted. All radiomics features were standardized using Z-score normalization to mitigate dimensional disparities.

### 2.6 Prognosis model

#### 2.6.1 Predictive task

Our predictive task aimed to accurately differentiate MM patients with deep response from those with non-deep response. To mitigate risks of bias and overfitting, we employed two methods. Firstly, we filtered features by employing the intraclass correlation coefficient (ICC) within and between observers, establishing a threshold of ICC > 0.70. Secondly, we applied the least absolute shrinkage and selection operator (LASSO) to the training dataset, using a five-fold cross-validation approach to identify the most predictive features. These strategies were employed to identify the most informative features while ensuring optimal predictive performance.

#### 2.6.2 Development and validation of the predictive model

We developed three models: the Clinical Model, the Radiomics Model, and the Combined Model. In the Clinical Model, using univariate logistic regression to analyze clinical data, combined with the actual clinical situation and the results of univariate logistic regression analysis, potential factors related to prognosis were included in the multiple logistic regression model. Clinical variables that may affect the evaluation of multiple myeloma treatment effectiveness were selected and used to construct the clinical model. All continuous features were normalized by Z score normalization. For clinical features, univariate (p < 0.1) and multivariate (p < 0.05) logistic regression were used to identify independent risk factors. Features with a p value lower than 0.05 were selected for inclusion in the corresponding clinical model.

Regarding the radiomics model, the features were extracted from CT and PET images, followed by Z-score standardization to reduce dimensional differences between different features, and cleaning of radiomics features to reduce the impact of outliers and missing values. To reduce bias and overfitting risks, feature selection steps included: first selecting features with intraclass correlation coefficients (ICC) greater than 0.70; then randomly selecting 20% of the dataset as independent test data, using Least Absolute Shrinkage and Selection Operator (LASSO) on the remaining data to select features with optimal predictive performance, used for constructing CT radiomics model and PET radiomics model. To maximize the recognition rate of radiomics algorithms, Logistic Regression (LR) machine learning classifier was used to build models with features selected by LASSO algorithm. Finally, combining CT and PET radiomics features selected by LASSO together, repeated the above process to build a joint radiomics model. Combine clinically selected risk factors with radiomics features to jointly build a comprehensive model for further evaluation of treatment efficacy in patients with MM.

To assess discrimination, we compared these models using six metrics: the area under the ROC curve (AUC), sensitivity, specificity, accuracy, precision, and F1 score on the test set. The calibration curve was assessed using the Hosmer-Lemeshow test by plotting the predicted ER probabilities against actual ER rates. The clinical utility of the models was evaluated using Decision Curve Analysis (DCA), which involves analyzing the net benefit of a range of threshold probabilities across the entire retrospective cohort. The performance of DCA is obtained by assessing the net benefit at various threshold probabilities. The best model was determined by comprehensive evaluation using Receiver Operating Characteristic (ROC) curves, decision curves, and calibration curves. Differences between the three models were compared using DeLong’s test, with a p-value less than 0.05 indicating significant differences between them. In summary, through comprehensive evaluation from both discrimination and calibration perspectives, we identified the optimal predictive model among the Radiomics Model, Clinical Model, and Combined Model for predicting postoperative outcomes in patients with MM.

### 2.7 Statistical analysis

To assess the normality of continuous features, we employed the Kolmogorov-Smirnov test. The T-test was used to compare variables with a normal distribution, which are represented as mean ± SD (standard deviation). For non-normally distributed data, the Mann-Whitney U test was used, and the data was represented using the median (inter-quartile range). Categorical variables were analyzed using either the chi-square test or Fisher’s exact test. The data was represented as counts (%). A p-value lower than 0.05 was considered statistically significant. The R software package (version 4.0.3) was used to process the demographic data for evaluating significant differences in the variables between the training and the validation set. Python (version 3.6) was employed for programming model training, validating the prediction model, as well as conducting statistical analysis.

## 3 Results

### 3.1 Assessment of clinic features

This study included a total of 165 patients (90 males: with a median age of 61 years). Patients had undergone two courses of treatment. Among them, 74 patients achieved DR. The treatment plan involved 133 patients within the training set and 32 patients within the test set. The baseline clinical characteristics, presented in **Table 1**, demonstrate consistency and comparability between the training and testing datasets. No statistically significant difference (p>0.05) was found in basic variables between the training set and the test set, including general characteristics (gender and age), medical history (hypertension, diabetes), and laboratory tests (BNP, etc). **Table 2** presents the outcomes of univariate and multivariate regression analyses assessing the association between various clinical features and the likelihood of achieving DR in patients with MM. After univariate analysis, Gender, Ca2+, and FL were significantly associated with the efficacy of treatment courses (p < 0.1); multivariate analysis revealed that Gender was an independent predictor of treatment efficacy, with a p-value of 0.041, and the risk ratio for males to females was 0.713(Using males as the reference category).

**Table 2:** Univariate and multivariate regression analysis of association between various clinical features and the likelihood of achieving DR in MM’s patients.

Parameters	Univariate analysis (p value)	Odds ratio (95% CI)	Multivariate analysis (p value)	Odds ratio (95% CI)
Age	0.547	1.099(0.807-1.496)		
Gender	0.093	0.767(0.563-1.044)	0.041	0.713(0.515-0.986)
Bone Marrow Plasma Cell	0.825	1.035(0.762-1.406)		
FL	0.062	1.372(0.984-1.914)	0.098	1.331(0.948-1.870)
EMD	0.491	0.898(0.660-1.221)		
HB	0.535	0.907(0.667-1.234)		
Cr	0.825	1.035(0.762-1.406)		
ALB	0.631	0.927(0.682-1.261)		
LDH	0.785	1.043(0.769-1.417)		
β2-MG	0.699	1.062(0.782-1.442)		
PLT	0.183	1.237(0.905-1.691)		
HCRP	0.545	1.101(0.807-1.503)		
Ca2+	0.077	1.335(0.970-1.838)	0.058	1.383(0.989-1.935)
DS-staging	0.514	1.109(0.813-1.513)		
ISS-staging	0.897	1.020(0.751-1.387)		
RISS-staging	0.603	1.085(0.798-1.475)		
Liver SUVmax	0.475	1.119(0.823-1.521)		
SUVmax	0.438	0.883(0.644-1.210)		
TLG	0.332	1.295(0.768-2.183)		
MTV	0.599	1.088(0.795-1.488)		

FL, focal lesion; EMD, extramedullary; HB, hemoglobin; Cr, creatinine; ALB, albumin; LDH, lactate dehydrogenase; β2-MG, β2-microglobulin; PLT, platelet; HCRP, hypersensitive C-reactive protein; DS staging, Durie Salmon staging; ISS, International Staging System; R-ISS, the revised International Staging System; SUVmax, max standardized uptake value; TLG, total lesion glycolysis; MTV, metabolic tumor volume.

### 3.2 Assessment of radiomic features

**Figure 4:**
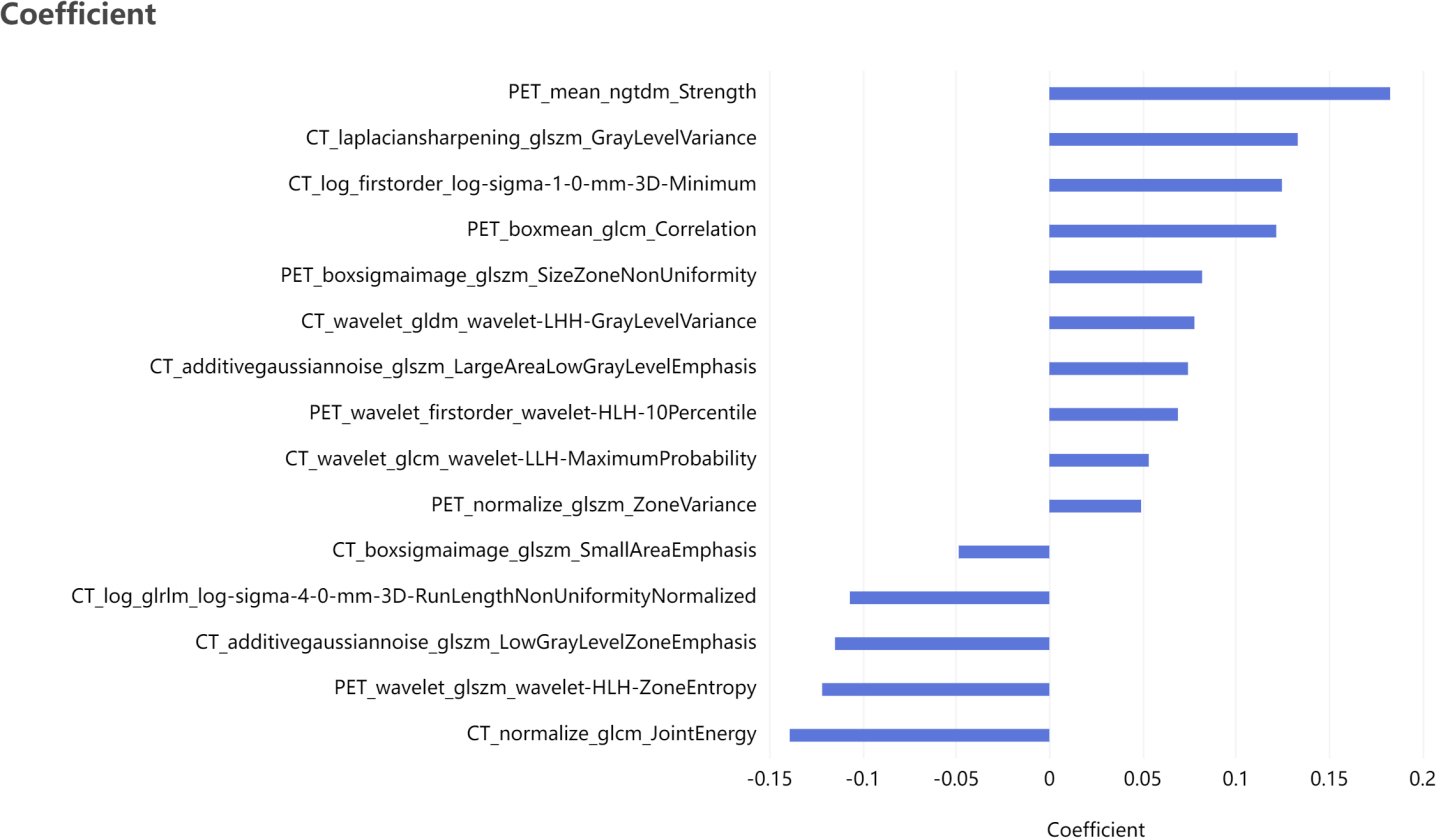
Bar plot of the selected features with non-zero coefficients, showing their relative contributions to the model.

Based on the labeled ROI, a total of 2264 features were extracted from each image of the two imaging modalities, namely PET and CT. Further refinement was conducted through consistency testing, resulting in 2034 and 2103 robust features for PET and CT, respectively. Following LASSO feature selection, 10 features were identified by the PET model, including 3 first-order features and 7 texture features, while 16 features were identified by the CT model, including 2 first-order features and 14 texture features. Utilizing these identified features, the PET-CT Radiomics Model was developed, incorporating 15 features in total, including 2 first-order features and 13 texture features. **Figure 4** present the selected features and their respective coefficients, which reflect each feature’s contribution to the model’s predictive performance. Specifically, the features comprise both intensity-based (first-order) and texture-based parameters from PET and CT images, collectively enabling accurate discrimination of patients with different therapeutic responses.

### 3.3 Comparison between different models

A radiomics model and a clinical model were constructed using logistic regression, utilizing radiomic features selected via LASSO and clinical features chosen through univariate and multivariate regression analyses, respectively. Furthermore, variables from both models were integrated to establish a combined model using logistic regression.

**Table 3**; **Figure 5** show the performance of different models in predicting DR in MM patients. Compared with the clinical model, the three models based on radiomics features demonstrated better predictive performance. In contrast to the PET model and the CT model, the PET-CT model yielded the best results with the AUC (95% CI), sensitivity, specificity, accuracy, and F1 score of 0.881 (0.82-0.94), 0.750, 0.822, 0.789, and 0.763 in the training set, and 0.809 (0.66-0.96), 0.714, 0.778, 0.75, and 0.714 in the test set, respectively.

**Table 3:** Predictive performance of different models for therapeutic effect in newly-diagnosed MM patients in training set and testing set.

Models	AUC^*^		Sensitivity		Specificity		Accuracy		F1 score	
	Train	Test	Train	Test	Train	Test	Train	Test	Train	Test
Clinical	0.568 (0.47-0.67)	0.556 (0.35-0.76)	0.533	0.500	0.603	0.611	0.571	0.562	0.529	0.500
PET	0.775 (0.70-0.85)	0.568 (0.37-0.77)	0.733	0.643	0.630	0.556	0.677	0.594	0.672	0.581
CT	0.837 (0.77-0.91)	0.742 (0.57-0.91)	0.733	0.643	0.767	0.833	0.752	0.750	0.727	0.692
PET + CT	0.881 (0.82-0.94)	0.809 (0.66-0.96)	0.750	0.714	0.822	0.778	0.789	0.750	0.763	0.714
PET + CT + Clinical	0.892 (0.84-0.95)	0.814 (0.67-0.96)	0.750	0.714	0.836	0.778	0.797	0.750	0.769	0.714

MM, Multiple Myeloma.

^*^AUC, area under the receiver operating characteristic curve.

**Figure 5:**
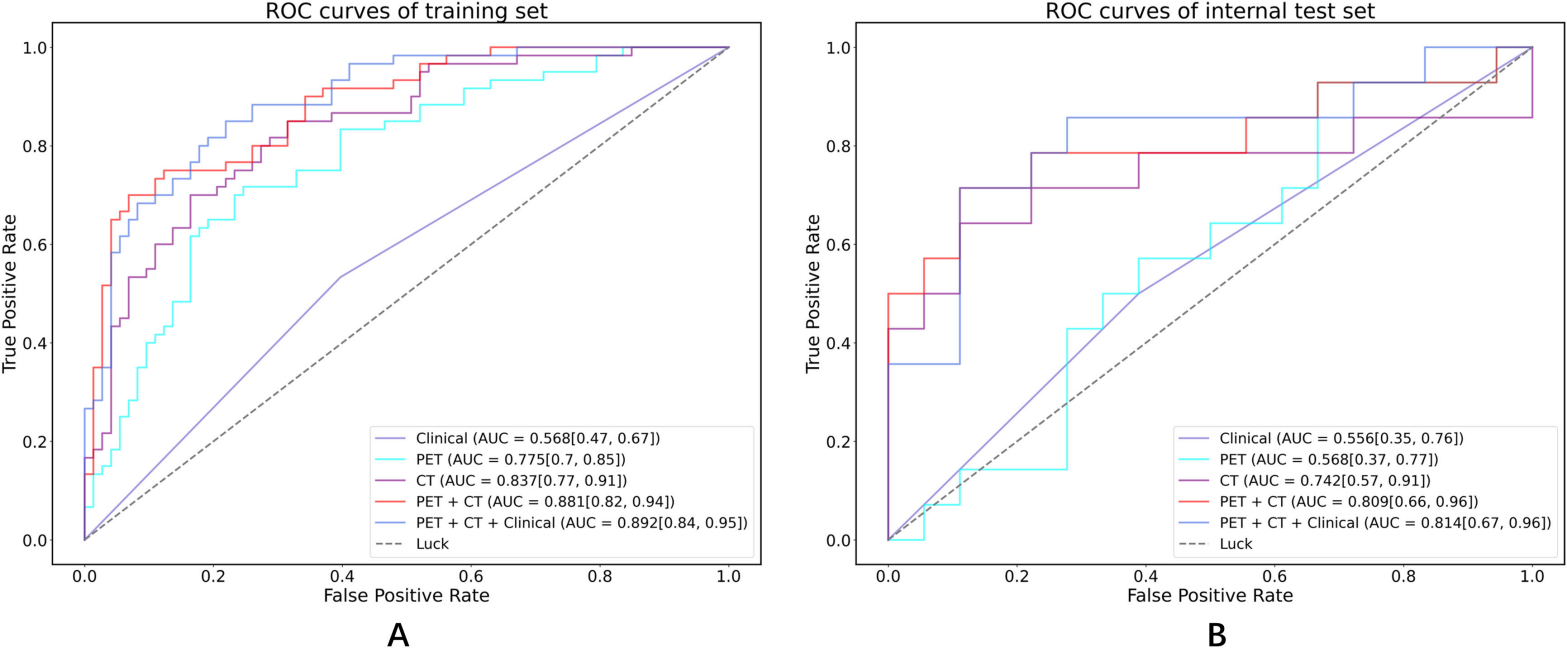
ROC curves in both the training set and internal test cohorts. **(A)** ROC curves of training set; **(B)** ROC curves of internal test set.

**Figure 6:**
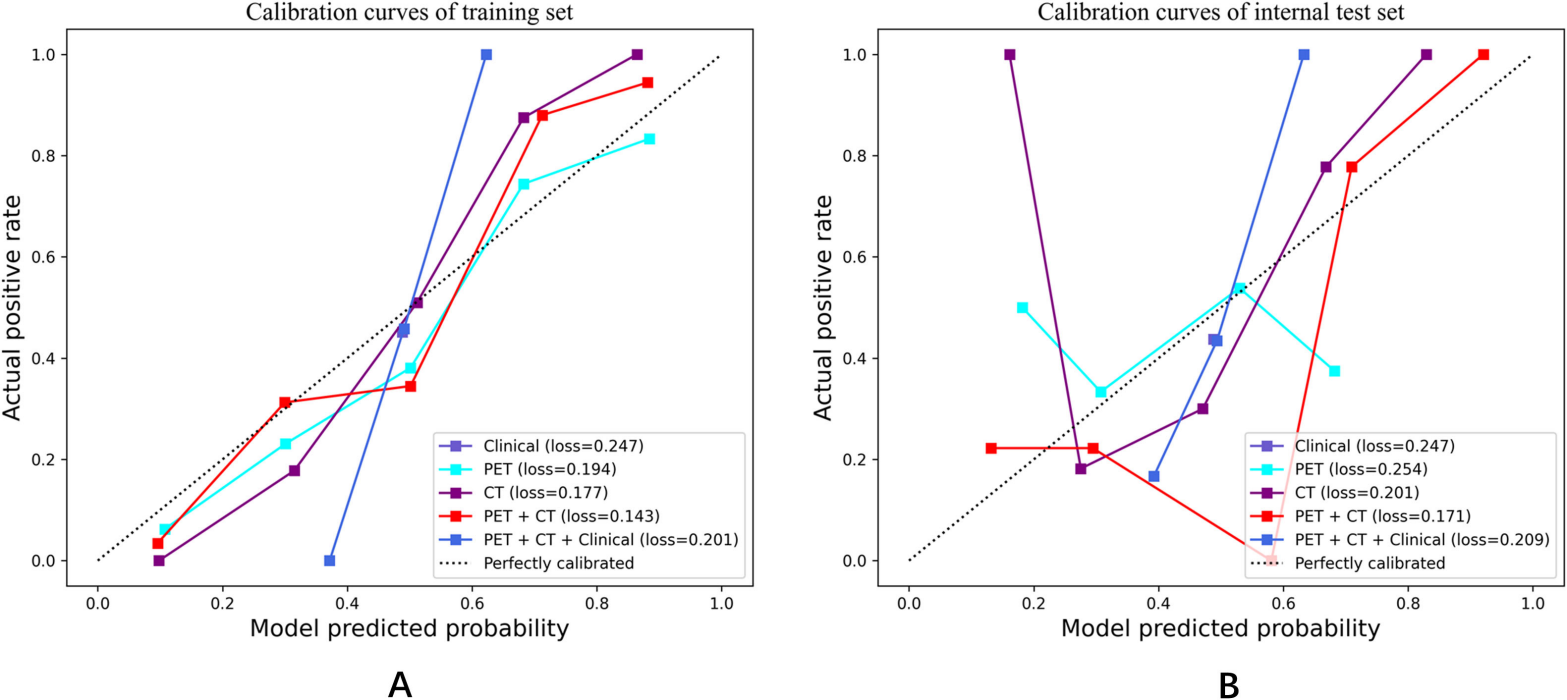
Calibration curves in both the training set and internal test cohorts. **(A)** Calibration curves of training set; **(B)** Calibration curves of internal test set.

**Figure 7:**
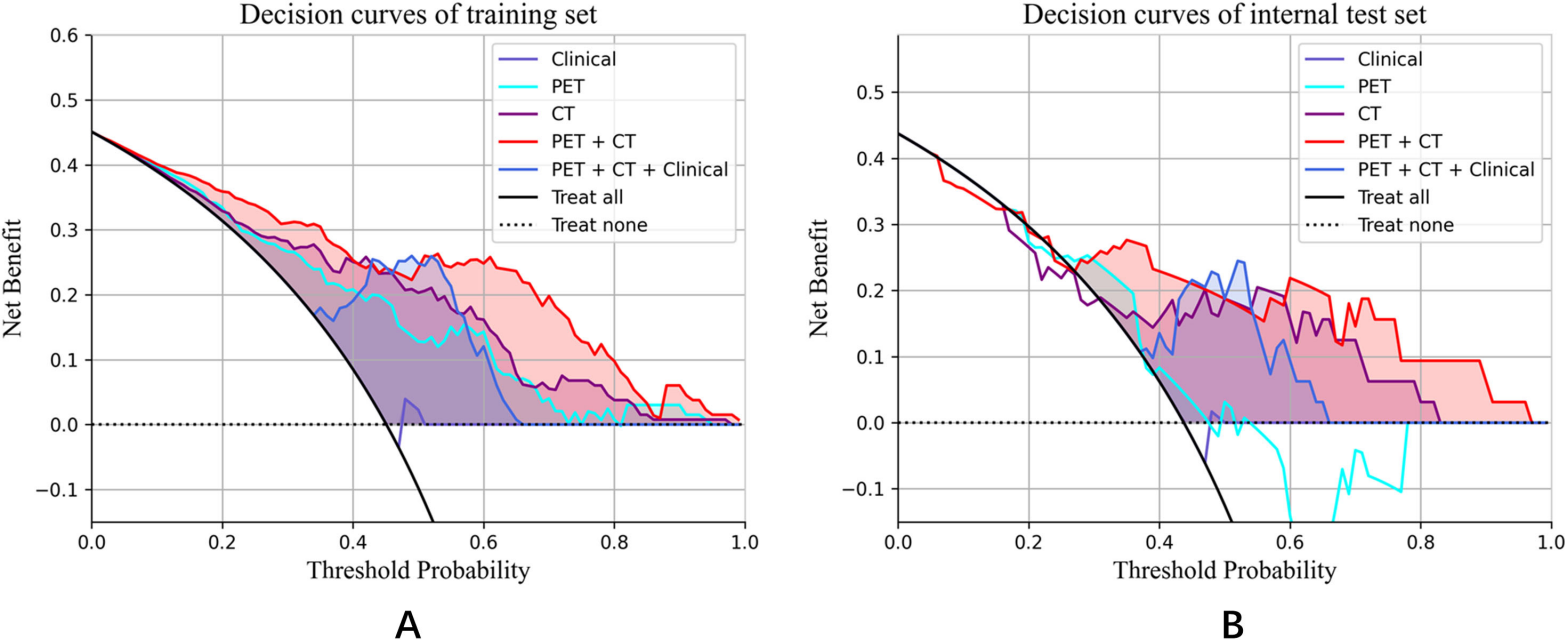
Decision curve analysis for the training set and internal test set. **(A)** Decision curves of training set; **(B)** Decision curves of internal test set.

Notably, the performance of the integrated model was further improved with the combination of clinical features and radiomic features with the AUC (95% CI), sensitivity, specificity, accuracy, and F1 score of 0.892 (0.84-0.95), 0.750, 0.836, 0.797, and 0.769 in the training set, and 0.814 (0.67-0.96), 0.714, 0.778, 0.750, and 0.714 in the test set, respectively (**Table 3**). From the results, the PET + CT radiomics model and the PET + CT + Clinical integrated model exhibited superior performance across all metrics. The calibration curves is shown in **Figure 6**. Upon comprehensive comparison of their decision curves (**Figure 7**), the PET + CT radiomics model’s calibration curve was closer to the diagonal, and it also had the largest area under the decision curve, indicating the overall optimal performance.

Furthermore, **Table 4** presents the pairwise comparison of prediction performances for different models by DeLong’s test. Both the PET + CT + Clinical model and the PET + CT model significantly outperformed the Clinical model and the PET model, indicating that the integration of multiple data sources could potentially enhance predictive performance although no significant difference was observed between the groups of PET + CT *vs.* PET model in the testing cohort, PET + CT + Clinical *vs.* CT, and PET + CT *vs.* CT, respectively.

**Table 4:** Comparison of model differences (DeLong test).

Models	Clinical		PET		CT		PET + CT		PET + CT + Clinical	
	Train	Test	Train	Test	Train	Test	Train	Test	Train	Test
Clinical	>0.999	>0.999	0.0014	0.9275	<0.0001	0.1983	<0.0001	0.0368	<0.0001	0.0131
PET	0.0014	0.9275	>0.999	>0.999	0.1328	0.3169	0.0003	0.0565	<0.0001	0.0451
CT	<0.0001	0.1983	0.1328	0.3169	>0.999	>0.999	0.1803	0.1817	0.1004	0.1789
PET + CT	<0.0001	0.0368	0.0003	0.0565	0.1803	0.1817	>0.999	>0.999	0.3116	0.9036
PET + CT + Clinical	<0.0001	0.0131	<0.0001	0.0451	0.1004	0.1789	0.3116	0.9036	>0.999	>0.999

## 4 Discussion

The precise prediction of MM patients who achieve deep responses holds considerable importance for directing treatment, monitoring disease progression, evaluating prognosis, and enhancing the quality of life of patients. In this investigation, we developed radiomics models that leveraged PET/CT features to assess therapeutic responses in individuals newly diagnosed with MM. The principal findings indicated that the radiomics model, which incorporated PET/CT features, significantly surpassed models based solely on clinical or PET characteristics. Furthermore, the amalgamation of multimodal data within the PET + CT and PET + CT + Clinical models yielded optimal predictive performance, achieving area under the curve (AUC) values of 0.809 and 0.813, respectively. These findings highlight the advantages of integrating multimodal data to enhance prediction accuracy.

In this study, we examined the influence of clinical characteristics on the prediction of patients with DR in MM. Surprisingly, only gender emerged as a significant predictor of therapeutic efficacy following multivariate logistic regression analysis, with a notably better prognosis for females. Indeed, there exists controversy regarding the impact of gender on the prognosis of MM. The majority of studies suggest that female patients with MM generally experience better prognoses compared to their male counterparts (30–33), which aligns with our observations. Potential explanations may include healthier attitudes and behaviors among women, resulting in greater participation in health-promoting activities post-treatment (34). Additionally, men often present with more comorbidities at the time of MM diagnosis, which could adversely affect survival rates. Biological factors may also play a role in these gender disparities in MM outcomes. For instance, hormone-related pharmacokinetic variations in lymphoma indicate that elevated rituximab serum levels in females correlate with improved progression-free survival (35). Moreover, a greater presence of regulatory T cells (Tregs) in males may contribute to their less favorable outcomes (36). Nonetheless, other studies have reported no significant influence of gender on prognosis (36). Our findings emphasize the necessity of accounting for gender differences in the management of MM and suggest that future research should delve deeper into the underlying biological and behavioral mechanisms to refine personalized treatment strategies.

To date, both ISS and RISS are widely utilized for risk stratification in MM. However, due to the heterogeneity of this disease, a singular system may not adequately capture the nuances applicable to all patients. For those with non-secretory or low-secretory MM, where the tumor burden is elevated alongside low serum 2-MG levels, ISS staging may prove inadequate. Additionally, certain patients displaying low serum β2-MG levels during early disease stages may present with chromosomal translocation t(4;14) and other cytogenetic anomalies associated with poor prognosis. Zhou H et al. demonstrated in their study that both RISS and ISS failed to significantly differentiate early relapse risk (37). Our multivariate analysis revealed that neither ISS nor RISS effectively distinguished patients with deep responses, indicating that conventional staging systems may not accurately reflect the intricate biological complexities of MM. A more holistic approach is warranted for evaluating tumor burden in patients diagnosed with MM.

Radiomics is an emerging and rapidly advancing domain within the realm of medical imaging, focusing on the analysis of medical images to extract high-dimensional quantitative data that unveils concealed information not readily apparent to the naked eye (15, 17). A range of medical imaging modalities can facilitate radiomic analysis in patients with MM, with MRI being the most extensively utilized, followed by CT and PET (38). In comparison to MRI and CT, PET provides a more insightful approach for assessing the metabolic activity of MM lesions, thereby offering distinct advantages in evaluating patient responses to therapy. This rationale underpins the IMWG recommendation of 18F-FDG PET/CT as the current “gold standard” for assessing and monitoring responses to anti-myeloma treatment (1, 2). However, differentiating between focal and diffuse patterns remains a considerable challenge. Consequently, several studies have turned their focus to radiomics methodologies, illustrating the potential applications of radiomics derived from PET/CT in differential diagnosis, MRD detection, and prognosis prediction for MM (21). Reports on the application of radiomics in predicting therapeutic efficacy for patients with newly diagnosed MM are notably scarce. In our investigation, models predicated on radiomic features demonstrated a substantial advantage over clinical models in identifying newly diagnosed patients with deep responses, exhibiting significantly higher values of AUC, sensitivity, specificity, accuracy, and F1 score (**Table 3**). This superiority is likely attributable to radiomics’ capacity to capture intricate details of tumor heterogeneity, offering insights that transcend traditional clinical features. Given the high heterogeneity of MM, quantitatively characterizing its inter- and intra-tumoral variations could significantly enhance prognostic assessments. Importantly, our findings indicate that the integration of multiple data modalities yielded the most robust predictive performance, as exemplified by the PET+CT and PET+CT+Clinical models. These models achieved superior accuracy in distinguishing between patients with deep responses and those without, suggesting their potential utility for risk stratification in MM. While our results do not directly evaluate treatment outcomes, they imply that such stratification may inform personalized therapeutic strategies in the future. For instance, patients exhibiting a deep response might be spared from unnecessary intensive treatment, whereas those without could benefit from closer monitoring and timely adjustments to their therapy. Furthermore, this study underscored prospective trends in merging radiomics with other data types, with the goal of comprehensively elucidating MM through the unveiling of complex connections between imaging phenotypes and the molecular mechanisms driving disease progression (38).

At present, the commonly used first-line regimens may include bortezomib, lenalidomide and dasatuzumab. For patients with poor efficacy after 2 courses of treatment, a new chemotherapy combination can be selected according to the patient’s drug resistance. For lenalidomide-resistant patients, proteasome inhibitors bortezomib, ixazomib or a new generation of proteasome inhibitors carfilzomib, a new generation of immunomodulators pomalidomide and CD38 monoclonal antibody-based regimens can be used. For bortezomib-resistant patients, regimens based on carfilzomib, pomalidomide, and CD38 monoclonal antibodies can be used. For patients with triple drug resistance, a regimen consisting of carfilzomib, pomalidomide, and celinesol can be used. If the patient’s age and physical condition allow, autologous hematopoietic stem cell transplantation can also be considered (39–42).

This study does possess certain limitations that warrant attention. First, the sample size is relatively small, necessitating validation through larger, multi-center datasets to ascertain the generalizability of our findings. Second, although cytogenetic testing was conducted for all patients, as our study was explicitly designed to concentrate on radiomics-based prediction. The prognostic significance of genetic alterations will be explored in a subsequent investigation. Third, the methodology was reliant on traditional radiomics techniques involving manual or semi-automatic feature extraction, which may introduce a degree of subjectivity. Future endeavors will investigate the integration of deep learning methodologies to facilitate automated feature learning, potentially enhancing predictive accuracy and thereby broadening the clinical applicability of radiomics in MM.

## 5 Conclusions

In conclusion, the radiomics model derived from FDG-PET/CT exhibits considerable promise for clinical implementation in forecasting profound treatment responses in patients newly diagnosed with MM. By non-invasively capturing tumor heterogeneity and metabolic attributes, this model serves as an objective and reproducible instrument for stratifying patients based on their probability of attaining a deep response. The amalgamation of radiomic characteristics with pivotal clinical factors, such as sex, further bolsters predictive precision, underscoring its applicability in personalized therapeutic strategies.

This model has the potential to facilitate early treatment assessment, inform adjustments, prevent overtreatment in responders, and pinpoint patients who require closer surveillance. Future multicenter validation and the incorporation of genetic markers may enhance its clinical relevance in the realm of precision medicine for MM.

## Data Availability

The original contributions presented in the study are included in the article/supplementary material. Further inquiries can be directed to the corresponding authors.
